# OD05 Frameworks For Synthesizing Qualitative Evidence In Health Technology Assessment: A Scoping Review

**DOI:** 10.1017/S0266462324001429

**Published:** 2025-01-07

**Authors:** Marilia Mastrocolla de Almeida Cardoso, Raphael Thomaz Marques, Juliana Machado-Rugolo, Lehana Thabane, Vilanice Püschel, Silke Anna Theresa Weber, Graciela Paula Duque, Rosimary Terezinha Almeida, Clarice Rodrigue, Sybelle Luzia Guimarães Drumond, Cristiane De Paula, Luciane Lopes, Mariana Gabriel, Meredith Vanston

## Abstract

**Introduction:**

Health technology assessment (HTA) agencies and researchers recognize the necessity of evidence-based methodologies beyond quantitative data to assess feasibility, appropriateness, meaningfulness, patient values, preferences, acceptability, and equity. Despite existing guidelines for synthesizing qualitative data, the HTA framework requires clarification. This review aims to describe the frameworks, tools, and processes used to synthesize qualitative evidence and assess the quality of HTA.

**Methods:**

Using the JBI methodology, the authors accessed databases such as MEDLINE, LILACS, CINAHL, Embase, Web of Science, Scopus, PsycINFO, Cochrane Library, JBI Database, and ScienceDirect. Grey literature searches included ProQuest, OpenGrey, CADTH’s Grey Matters, Google Scholar, and HTA agencies’ websites. Inclusion criteria focused on synthesizing qualitative evidence frameworks, methods for evidence synthesis, and quality rating. The review had a global scope, without specific population and time restrictions. Data, encompassing fundamental concepts, frameworks, methods, subjects, and objectives, were presented in tables and figures.

**Results:**

Out of 2,054 articles, 31 were included, mainly from Europe, with a predominant “guide” authored by an HTA agency and university. The majority of documents did not originate from agencies. Only three agencies developed specific documents. A surge in publications occurred in 2018/2019. Qualitative data in HTA were justified for opinions, acceptability, feasibility, and equity. SPICE was the most cited acronym; RETREAT was the preferred framework. Thematic synthesis was the most cited method, CASP for quality assessment. GRADE-CERQual graded evidence quality, and ENTREQ was cited for reporting qualitative research. The GRADE EtD framework was the sole tool mentioned for recommendations.

**Conclusions:**

This review highlights a growing trend in including qualitative evidence in HTA. While various proposals suggest instruments and methods, few documents cover all necessary steps, resulting in diverse recommendations. Standardizing processes can improve decision-making by guiding the integration of qualitative evidence, potentially enhancing recommendation quality. This ensures evidence on feasibility, appropriateness, significance, patient values, preferences, acceptability, and equity are considered.

